# Prevalence of patients harboring temporomandibular disorders in an otorhinolaryngology departament

**DOI:** 10.1016/S1808-8694(15)30105-1

**Published:** 2015-10-19

**Authors:** Alexandra Magalhães Silveira, Pedro Paulo Feltrin, Rachel Virgínia Zanetti, Mário Cláudio Mautoni

**Affiliations:** a M.S. DDS.; b PhD. DDS. Professor of the Master’s Degree Program in Dental Prosthesis - Centro de Pesquisa São Leopoldo Mandic - Advisor of the Master’s Degree Dissertation.; c Phd. DDS. Professor of the Master’s Degree Program in Dental Prosthesis - Centro de Pesquisa São Leopoldo Mandic.; d PhD. DDS. Full Professor of Semiology - UNISANTA, Santos, SP.; Centro de Pesquisas Odontológicas São Leopoldo Mandic.

**Keywords:** temporomandibular joint, temporomandibular disorders, orofacial pain, epidemiology, prevalence

## Abstract

The interaction between Temporomandibular disorders (TMD) and otalgia is, even nowadays, a reason for speculation and hypotheses raising. Several researchers suggest causes, consequences and alleged treatments.

**Study design:**

This is an epidemiological, sectional cohort study of prevalence.

**Aim:**

The study demonstrates the prevalence of patients harboring TMDs in an otorhinolaryngology department.

**Material and methodos:**

During a two-month period, 221 patients from the Otorhinolaryngology Department of the Hospital da Cidade de Passo Fundo, State of Rio Grande do Sul, Brazil were analyzed. A previously validated questionnaire was applied for data collection.

**Results:**

In the present study, the need for dental assessment was observed in 48 patients (21.72%). In this group there were 35 female (72.9%) and 13 males (27.1%). Only 15 patients (7.24%) were entirely free of TMD symptoms. The remaining patients reported the following TMD symptoms: headaches: 34.39%, neck and shoulder pain: 28.50%, pain on the ear region: 30.32% and joint noises in 23.98%.

**Conclusion:**

The prevalence of Temporomandibular disorders was 21.72%, being significantly higher among female subjects (p:0.0001). The prevalence regarding the indexes, was: TMD absent: 37.56%; mild TMD: 40.72%; moderate TMD 19% and severe TMD: 2.72%.

## INTRODUCTION

According to the American Academy of Temporomandibular Disorders, “Temporomandibular Disorders or Dysfunctions (TMD)”, is a collective term that encompasses many clinical problems involving the masticatory muscles, the temporomandibular joint (TMJ) and associated structures or both”. According to the classification of the International Headache Society (IHS-1987), TMD is a distinctive subgroup of muscle-skeletal and rheumatologic disorders of the orofacial region; and orofacial pain is item # 11 in the general classification of headaches.^1^

TMD signs and symptoms are very common in the population. Epidemiological research shows that more than 50% of the population presented at least one or more signs of TMD^2^, ^3^, ^4^; however, these figures are not translated into treatment necessity; it is estimated that only 3.6% to 7% of these individuals need some kind of intervention.^5^, ^6^, ^7^

One review of 18 epidemiological studies reports that the most common symptoms among patients with TMD are: TMJ sounds (19%); mandible stiffening and tiredness (11%); pain during mandibular function (6%); limitation of mandibular movements (8%), mandibular locking (4%), headaches (17%) and otalgias.^2^, ^3^

If we consider only the otorhinolaryngological symptoms of patients with TMD, we have the following picture: otalgia, present in 75% of the patients; hypoacusis, in 15%; nausea in 10%; vomit in 10%; ear fullness in 17.5%; tinnitus in 17.5% and autophonia in 15%.^9^

Otological symptoms and TMD association is already established, many are the researchers that suggest causes, consequences and alleged treatment. For sure, the most famous of these researchers was Costen^10^, who in 1934 published a treaty that, besides showing this association between the stomatognatic system and otalgia, formulated a theory on teeth, or the lack of them, by a cascade effect that triggered otological alterations. Before and after Costen, there were theories such as the one from Prentiss^11^, who in 1918 created the theory on the mechanical displacement, or the one from Vass^12^, who, in 1997, found evidence that the trigeminal ganglion was related to the cochlea, being responsible for the innervation of the blood vessels by it, thus having a possible role in the balance of normal vascularization and in some inner ear disorders. Throughout history, all these theories, and many others, were heavily criticized, and often times, partially reaccepted. The fact of the matter is that, until current days, there is still the doubt about the existence of some causal relationship between TMD and otological alterations.

Facing the numerous theories that relate TMD as a cause for otalgia, or the multiple otological signs and symptoms present in TMD, it is pertaining to investigate the percentage of patients with TMD in the otorhinolaryngology ward.

## MATERIALS AND METHODS

This research was of the descriptive type, with a cross-sectional sample, made up of 221 patients, from both genders, who went to the Otolaryngology ward of the Hospital da Cidade, in Passo Fundo, Rio Grande do Sul, during two months (July 1st to September 30th). This place is a center of medical reference in the region and encompassed 55 towns.

This research project has the approval protocol #: 05/140; São Leopoldo Mandic Postgraduate Studies Center, resolution #: 196/1.996 from the CNS-Ministry of Health, meeting held on May 20, 2005.

Exclusion criteria were:

- Patients below 18 or above 80 years of age: because of legal aspects regarding authorization to include them in the research and the fact that, the average age of men and women with TMD is of 32.2 and 33.8 years, respectively;^13^
- Patients who had suffered accidents and/or surgical intervention on their faces in the six months prior to the study, because they would still be under treatment;
- Patients with physical or mental disabilities that could affect judgment or filling out the questionnaire;
- Patients with autoimmune or degenerative disorders that could mask results.;
- Patients with history of chronic otitis or previous otological surgery.

Data for patient exclusion were checked through the patient’s medical chart.

As inclusion criteria we considered all the patients who came to the Otorhinolaryngology Ward during a certain period, who accepted to participate in the study.

### Questionnaire

For data capture, we used a self-applicable history questionnaire, without the interference of the researcher, aiming at detecting TMD. The questionnaire model, as well as its interpretation were validated by Fonsêca et al.^14^; other authors, such as Conti et al.^15^ and Conti^16^ also used it in their studies. For organizational reasons, patients also filled out a form with their personal information.

The TMD assessment questionnaire had ten questions. (Table 1)

**Table 1:** Questionnaire used to check for TMD.

HISTORY		YES	NO	SOMETIME
1.	Do you feel difficulties to open your mouth?	( ) Y	( ) N	( ) ST
2.	Do you feel difficulties in moving your mandible sideways?	( ) Y	( ) N	( ) ST
3.	Do you feel discomfort or muscle pain when chewing?	( ) Y	( ) N	( ) ST
4.	Do you have frequent headaches?	( ) Y	( ) N	( ) ST
5.	Do you feel neck and/or shoulder pain?	( ) Y	( ) N	( ) ST
6.	Do you feel pain in your ear, or near it?	( ) Y	( ) N	( ) ST
7.	Do you perceive any TMJ noise?	( ) Y	( ) N	( ) ST
8.	Do you consider your bite “abnormal”?	( ) Y	( ) N	( ) ST
9.	Do you use only one side of your mouth to chew?	( ) Y	( ) N	( ) ST
10.	Do you feel pain in your face when you wake up?	( ) Y	( ) N	( ) ST

#### CLASSIFICATION

The questionnaire’s interpretation as to TMD presence and classification was:

- For each “yes” answer, a score of “2” was assigned, “sometimes” had a score of “1”; and “no”, a score of “0”;
- For questions 6 and 7, if the symptoms were bilateral, “1” more point was added to the total value;
- Also for question 4, “1” more point was added when pain, besides frequent, was also intense.

The summation of the values obtained allowed us to classify the sample as far as TMD is concerned, and from now on it is considered as TMD index.

- values from 0 to 3: no TMD;
- values from 4 to 8: mild TMD;
- values from 9 to 14: moderate TMD;
- values from 15 to 23: severe TMD.

## RESULTS

When we analyzed patient distribution in relation to TMD indexes proposed for this study, we obtained the results described on Table 2.

**Table 2:** Interpretation of results in relation to TMD indexes.

TMD index	Number of individuals	Percentage
No TMD	83	37.56%
Mild TMD	90	40.72%
Moderate TMD	42	19%
Severe TMD	06	2.72%

Also the individuals considered as needing referral/treatment, in other words, as moderate and severe, the number found was of 48 patients (21.72%). Of these, 42 had moderate index; and 6 had severe index.

The relationship between need for treatment and gender was that: of the 48 patients who needed treatment, 35 were females (72.9%) and 13 were males (27.1%).

## DISCUSSION

Our research focused on a number of individuals who came to your otolaryngology ward, in other words, a specific population, and data collection was carried out by means of a self-applied questionnaire. Therefore, only the individuals’ symptoms were taken into account. The parameter, both for data interpretation as for need for treatment, was the same used by 14, Conti et al.^15^ and Conti^16^, in other words, the patients who obtained moderate to severe history index were considered for TMD assessment /treatment.

Considering symptoms prevalence, according to Greene^17^, there are four, so called classic symptoms in patients with TMD, commonly called the TMD’s cardinal signs. They are:

1- pain,
2- joint click sounds or others,
3- limitation in mandibular movements and
4- tenderness in the masticatory and/or neck muscles. Otological alterations such as vertigo and tinnitus may also be present.

The most frequent symptom, according to our sample, was headache, since 34.39% of the individuals complained of frequent headaches, and 33.48%, said they sometimes had it. It is interesting to notice that 3.17% of the ones who had frequent headache described it as very intense. This data is in agreement with D’Antonio et al.^18^, who, assessed patients in a similar target population, in other words, patients who went to an otorhinolaryngology ward, described headache as the most frequently found symptom in patients with TMD; however, in his study, the percentage of patients with it was of 54.4%. This apparent disagreement may have happened due to the difference in interpretation and the data collection method, since in the method hereby used, the patients who had occasional headache (34%) were not considered as having headaches.

In relation to the other symptoms, this investigation found as the second most common symptom, a constant pain in the neck and shoulders, affecting 28.51%, and, occasionally, in 35.75% of the individuals. As a third symptom, we had pain in the ear region or near it, with 30.32% of “yes” and 15.84% of “sometimes” answers. Joint noises came in fourth place, with 23.98% of the interviewees describing it as constant and 17.19% as occasional.

The literature is broad in the description of symptoms. Now, considering only the number of symptoms found, the present investigation found that only 7.24% were totally free from them, and all the other individuals reported at least one symptom.

In a general population, Agerberg and Inkapööl5, with 637 persons from 18 to 65 years, found that only 12% were completely signs and symptoms free; nonetheless, De Kanter et al.^19^, assessing an adult population from Holland, found 1/5 of individuals with one or more symptoms of dysfunction.

The issue that females have more TMD than males has always been a very controversial topic.

When they analyzed asymptomatic populations, authors such as Agerberg and Carlsson^20^, Helkimo^21^, ^22^, ^23^, Salonen et al.^4^, De Kanter et al.^19^, Lipton et al.^24^, Pow et al.^25^ e Bomjardim et al.^26^ considered the difference in signs and symptoms between both genders not to be statistically significant; however headache frequencies was very much present in women.

Nonetheless, when considering one specific population of patients, differences between genders are more significantly altered.

The results we attained with our study were: of the 83 patients who did not have TMD (according to the index), 37 (16.74%) were females and 46 (20.82%), were males; of the 90 patients with mild TMD, 53 (23.98%) were females and 37 (16.74%), males; patients with moderate index (42 persons), 13.12% were females and 5.88% were males; and, of the 6 patients who had severe TMD index (2.72%), they were all females. It is also pertaining to highlight that, of the patients who required treatment, that is, moderate to severe TMD index, 72.9% were females and only 27.1% were males.

These data are similar to the ones found in the literature. Britto9, when assessing otological alterations in patients with temporomandibular disorders who went to the TMD ward of the Dental School of the UFRJ, concluded that the frequency of TMD is higher among females, and 92.5% of the patients with TMD who sought specialized care were females.

Such data is corroborated by Agerberg and Carlsson^27^, Conti et al.^15^, Conti^16^, Monteiro et al.^28^ and Bove et al.^28^ who also describe in their conclusions that they found a significantly higher prevalence of TMD in women.

Although TMD prevalence difference between the genders is yet to be thoroughly understood, some theories have tried to explain why women seem to be more affected than men, as Agerberg and Sandstrom^30^, believe that women are not so skilled to deal with pressure, causing a greater number of functional disorders. Abubaker et al.^31^ shows that estrogen receptors in women’s TMJ are present in greater quantity. Le Resche et al.^32^ associated this to the possibility of exogenous hormones being an important cause of difference between the genders.

Despite such evidences, the true reason, or the whole set of them, of women more frequently seeking specialized care for TMD is still unknown and requires further studies.

TMD’s epidemiology is very complex in turning prevalence values into the need for treatment, and such fact has generate and still generates much criticism; what is known for sure is that, even with different philosophies and lines of thought within the field of TMD, prevalence values for signs and symptoms can not be translated into treatment need. Nonetheless, because of numerous variables, it is difficult to perform specific studies regarding the need for TMD treatment.

The present investigation obtained a total of 21.7% individuals who were considered to require treatment, in other words, who obtained a moderate to severe history index, of which 19% were moderate and 2.7% were severe cases. Such data is in agreement with the findings from D’Antonio et al.18 (p: 0.108), who noticed the presence of TMD in 17.2% of the 523 patients interviewed in an otorhinolaryngology ward.

Our findings are also in agreement with the first epidemiologic studies of a general population, as one of the classic studies of Helkimo et al.^33^ which suggests that from 20 to 25% of the individuals studied needed treatment. Nonetheless, when we analyze the first TMD epidemiological studies, we see that the interpretation methodology was different from the one applied by D’Antonio et al’s team.18 and by the present investigation, since the researchers believed that treatment need was relatively important. Today, the trend is towards an interpretation of milder signs and symptoms in need for treatment, in whom the benefit potential stemming from treatment is, often times, the very treatment guideline. Thus, we could not compare similar studies because of different interpretations, and this brings about much confusion at the time of analyzing results.^33^

Considering how complex the topic is, the diversity of methodologies and the target population, the interpretation of this result remains in the open, requiring further studies that use similar protocols.

## CONCLUSION

Facing the results attained and statistically analyzed, we may conclude that: the prevalence of severe and moderate TMD was of 21.72%; the prevalence of TMD was significantly higher in females (p: 0.0001); and the prevalence found in relation to the indices was: no TMD: 37.56%; mild TMD: 40.72%; moderate TMD: 19%; severe TMD: 2.72%.

## References

[1] McNeill CH (1993). The American Academy of Orofacial Pain.

[2] Okenson JP (1998). Dor orofacial, guia de avaliação, diagnóstico e tratamento.

[3] Carlsson GE (1884). Epidemiological studies of signs and symptoms of temporomandibular joint-pain-dysfunction. Aust Prosthodont Soc Bull.

[4] Salonen L, Hellden L, Carlsson GE (1990 Fall). Prevalence of signs and symptoms of dysfunction in the masticatory system: an epidemiologic study in an adult Swedish population. J Craniomandib Disord.

[5] Agerberg G, Inkapool I (1990 Summer). Craniomandibular disorders in an urban Swedish population. J Craniomandib Disord.

[6] Paiva G, Nasr MK, Paiva AF (1997). Características de pacientes portadores de desordem temporomandibular: Avaliação de 400 casos. ATM Scientia.

[7] McNeill C, Mohl ND, Rugh JD (1990 Mar). Temporomandibular disorders: diagnosis, management, education, and research. J Am Dent Assoc.

[9] Britto LH (1998). Alterações otológicas nas desordens temporomandibulares. Rev Bras Otorrinolaringol.

[10] Costen JB (1997). A syndrome of ear and sinus symptoms dependent upon disturbed function of the temporomandibular joint. 1934. Ann Otol Rhinol Laryngol.

[11] Prentiss H (1918). A preliminary report upon the temporomandibular articulation in the human type. Dent Cosm.

[12] Vass Z, Shore SE, Nuttall AL (1997). Trigeminal ganglion innervation of the cochlea–a retrograde transport study. Neuroscience.

[13] Luz JG, Oliveira NG (1994). Incidence of temporomandibular joint disorders in patients seen at a hospital emergency room. J Oral Rehabil.

[14] Fonsêca DM (1992). Disfunção Craniomandibular (DCM) - diagnóstico pela anamnese. FOB - Faculdade de Odontologia de Bauru.

[15] Conti PC, Ferreira PM, Pegoraro LF (1996 Summer). A cross-sectional study of prevalence and etiology of signs and symptoms of temporomandibular disorders in high school and university students. J Orofac Pain.

[16] Conti ACCF (2000). Avaliação transversal da relação entre sinais e sintomas das disfunções temporomandibulares e o tratamento ortodôntico. FOB - Faculdade de Odontologia de Bauru.

[17] Greene CS, Lerman MD, Sutcher HD (1969). The TMJ pain-dysfunction syndrome: heterogeneity of the patient population. J Am Dent Assoc.

[18] D’ Antonio W, Ikino C, Castro S (2000). Distúrbio temporomandibular como causa de otalgia: um estudo clínico. Rev Bras Otorrinolaringol.

[19] De Kanter RJ, Kayser AF, Battistuzzi PG (1992). Demand and need for treatment of craniomandibular dysfunction in the Dutch adult population. J Dent Res.

[20] Agerberg G, Carlsson GE (1973). Functional disorders of the masticatory system. II. Symptoms in relation to impaired mobility of the mandible as judged from investigation by questionnaire. Acta Odontol Scand.

[21] Helkimo M (1974). Studies on function and dysfunction of the masticatory system. I. An epidemiological investigation of symptoms of dysfunction in Lapps in the north of Finland. Proc Finn Dent Soc.

[22] Helkimo M (1974). Studies on function and dysfunction of the masticatory system. II. Index for anamnestic and clinical dysfunction and occlusal state. Sven Tandlak Tidskr.

[23] Helkimo M (1974). Studies on function and dysfunction of the masticatory system. IV. Age and sex distribution of symptoms of dysfunction of the masticatory system in Lapps in the north of Finland. Acta Odontol Scand.

[24] Lipton JA, Ship JA, Larach-Robinson D (1993). Estimated prevalence and distribution of reported orofacial pain in the United States. J Am Dent Assoc.

[25] Pow EH, Leung KC, McMillan AS (2001 Summer). Prevalence of symptoms associated with temporomandibular disorders in Hong Kong Chinese. J Orofac Pain.

[26] Bonjardim LR, Gavido MBD, Pereira LJ (2005). Sinais e sintomas de disordens temporomandibular em adolescentes. Braz Oral Res.

[27] Agerberg G, Carlsson GE (1975). Symptoms of functional disturbances of the masticatory system. A comparison of frequencies in a population sample and in a group of patients. Acta Odontol Scand.

[28] Montero X, Badía C, Rojas JC (2004). Otalgia en pacientes con transtorno funcional doloroso temporomandibular. Rev Otorrinolaring Cir Cabeza Cuello.

[30] Agerberg G, Sandstrom R (1998 Feb). Frequency of occlusal interferences: a clinical study in teenagers and young adults. J Prosthet Dent.

[31] Abubaker AO, Raslan WF, Sotereanos GC (1993). Estrogen and progesterone receptors in temporomandibular joint discs of symptomatic and asymptomatic persons: a preliminary study. J Oral Maxillofac Surg.

[32] LeResche L, Saunders K, Von Korff MR (1997). Use of exogenous hormones and risk of temporomandibular disorder pain. Pain.

[33] Helkimo MI, Bailey JO, Ash MM (1979). Correlations of electromyographic silent period duration and the Helkimo dysfunction index. Acta Odontol Scand.

